# 深度学习在质谱成像数据分析中的应用研究进展

**DOI:** 10.3724/SP.J.1123.2023.10035

**Published:** 2024-07-08

**Authors:** Dongdong HUANG, Xinyu LIU, Guowang XU

**Affiliations:** 1.中国科学院大连化学物理研究所,中国科学院分离分析化学重点实验室,辽宁省代谢组学重点实验室, 辽宁 大连 116023; 1. CAS Key Laboratory of Separation Science for Analytical Chemistry, Dalian Institute of Chemical Physics, Chinese Academy of Sciences, Liaoning Province Key Laboratory of Metabolomics, Dalian 116023, China; 2.大连理工大学化学学院,辽宁 大连 116024; 2. College of Chemistry, Dalian University of Technology, Dalian 116024, China

**Keywords:** 质谱成像, 深度学习, 神经网络, 数据分析, mass spectrometry imaging (MSI), deep learning, neural network, data analysis

## Abstract

质谱成像(MSI)是一种用于表征化合物空间分布特征的方法。随着采集方式的多样化发展和灵敏度等的不断提高,该方法产生的数据总量和分析复杂度呈指数增长,给数据后处理带来了诸多挑战。深度学习(DL)是一种在数据分析和图像识别中广泛应用的强大工具,对于质谱成像数据分析具有巨大潜力。本文综述了深度学习在质谱成像数据分析中的研究现状、应用进展和面临的挑战,重点涵盖数据预处理、图像重构、聚类分析和多模式融合4个核心阶段;还列举说明了深度学习与质谱成像技术相结合在肿瘤区域划分和亚型诊断等研究中的高效应用。本综述对该研究方向未来的发展趋势进行了展望,旨在促进人工智能与质谱分析更好的结合。

## Abstract (en)

Mass spectrometry imaging (MSI) is a promising method for characterizing the spatial distribution of compounds. Given the diversified development of acquisition methods and continuous improvements in the sensitivity of this technology, both the total amount of generated data and complexity of analysis have exponentially increased, rendering increasing challenges of data postprocessing, such as large amounts of noise, background signal interferences, as well as image registration deviations caused by sample position changes and scan deviations, and etc. Deep learning (DL) is a powerful tool widely used in data analysis and image reconstruction. This tool enables the automatic feature extraction of data by building and training a neural network model, and achieves comprehensive and in-depth analysis of target data through transfer learning, which has great potential for MSI data analysis. This paper reviews the current research status, application progress and challenges of DL in MSI data analysis, focusing on four core stages: data preprocessing, image reconstruction, cluster analysis, and multimodal fusion. The application of a combination of DL and mass spectrometry imaging in the study of tumor diagnosis and subtype classification is also illustrated. This review also discusses trends of development in the future, aiming to promote a better combination of artificial intelligence and mass spectrometry technology.

质谱成像(mass spectrometry imaging, MSI)^[1,2]^是一种能够同时获取化合物质量、相对含量和空间分布信息的分析技术。它结合了质谱技术和成像技术,通过将样本表面划分成微小区域,再逐个区域进行质谱采集,得到每个区域的化学成分信息。经过可视化后,绘制出化合物在整个区域中的分布图像,实现对目标化合物的检测和定位。MSI具有广泛的应用领域,在生物医学研究中,通过空间分辨代谢组学分析生物样本中的代谢产物^[3,4]^、肿瘤标志物^[5]^和暴露标志物^[6]^等的空间分布以及变化情况,从而提供关于生物过程、毒性效应和疾病发展等的重要信息,为细胞生物学、病理学等提供支持;在药物研发中,MSI可以提供药物在组织中的分布信息^[7,8]^,明确药物作用靶点,提高药物疗效;在植物科学^[9]^和食品科学领域^[10]^,该技术被用于研究植物代谢途径、有效成分的分布等。

近年来,MSI技术发展迅速^[11]^,通过改造质谱仪的设计和优化质谱图像的采集条件,实现了更高的空间分辨率和质谱分辨率^[12,13]^;除了传统的成像模式,如正离子和负离子模式,还发展了其他成像模式,如离子碎裂成像、同位素标记成像、化学反应动力学成像等,这些模式提供了更全面的信息来解析不同的化学组分和反应过程;将MSI与光学成像、电子成像相结合的多模态融合分析可获得不同尺度和信息的综合成像结果,进一步提高解析度和信息丰富性^[14,15]^。

自动化流程和并行数据采集使MSI向着大规模和高通量方向发展,也为其数据后处理带来诸多挑战:①大量噪声和背景信号(如仪器噪声、化学噪声、基质效应等)的存在会降低信噪比,对精准的分子鉴定和分布分析造成干扰,需要开发出有效的噪声去除和背景校正方法,以提高数据的质量和可靠性;②样本的位置变化和扫描偏移等因素会导致图像配准和畸变校正的问题,需要开发新的图像配准算法和技术,确保相邻切片、不同样本或不同成像时间点之间的图像对齐和一致性;③通过有效的数据归一化和标准化消除样品间和区域间的强度差异;④将复杂数据可视化成清晰直观的图像,提取有价值的特征用于高精度的聚类、ROI(region of interest)划分等分析,进一步与其他数据模态进行整合和解释,也是一个重要挑战。

深度学习(deep learning, DL)^[16]^是一种特殊的机器学习(machine learning, ML)方法,通过构建和训练神经网络模型,对数据和图像中的特征进行自动提取,并运用数据增强、特征提取和模式识别等技术完善模型,通过迁移学习的方式分析目标数据,以此实现全面和深入的分析,在数字和图像等数据的处理中表现出强大的功能,极大地提高了研究的分析效率和可解释性。本文将系统地介绍DL在MSI数据分析中的方法发展以及结合DL的MSI方法在肿瘤诊断研究中的应用。

## 1 基于DL的MSI数据分析方法

MSI作为无标记高通量表征分子分布的强大技术,其数据处理和分析过程较为复杂,图1显示了MSI数据分析的基本流程,包括数据预处理(data preprocessing)、图像重构(image reconstruction)、聚类分析(cluster analysis)和多模态融合(multimodal fusion)。相较于传统ML方法,DL能以端到端的方式自动学习复杂的特征、深层挖掘大规模数据。

**图1:**
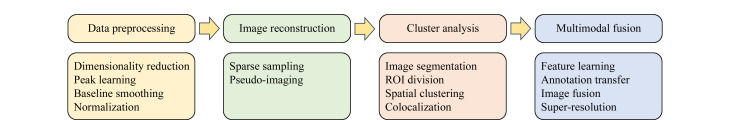
MSI数据分析的基本流程

### 1.1 数据预处理

新一代质谱技术能从单个组织切片样本产生大体量数据,需要进行复杂的数据预处理,包括峰提取、偏移校准、基线平滑、响应归一化等,流程化处理通常由软件执行,例如,BRUKER公司开发的商用软件SCiLS Lab以及He等^[17,18]^自主研发的软件MassImager等。然而,预处理后的数据维数仍然较高,需要引入降维技术分析数据;传统的降维方法如主成分分析(principal component analysis, PCA)、线性判别分析(linear discriminant analysis, LDA)、非负矩阵分解(non-negative matrix factorization, NMF)^[19]^和*t*分布随机近邻嵌入(*t*-distributed stochastic neighbor embedding, *t*-SNE)^[20]^等,多基于线性监督原理,即以一定的线性假设为前提,认为数据在低维空间中呈现线性结构,这导致在降维过程中丢失部分信息,造成原始数据的复杂性和差异性无法完全呈现。

基于非线性降维原理的DL模型可以更好地处理非线性结构和高维度的MSI数据,2016年,Thomas等^[21]^首次将DL引入到MSI数据的降维处理中,使用自编码器(autoencoder, AE)对大鼠大脑横切面的基质辅助激光解吸(MALDI)-MSI数据集进行处理,通过提取核心特征并整合到可控数量的隐藏节点中实现降维。该模型能捕获图像所有区域的数据,未出现丢失信息,且通过与降维空间的固定映射,实现了多个数据集的直接比较。Inglese等^[22]^建立了一种基于AE结合*t*-SNE的非线性参数化降维方法来划分肿瘤区域,该方法将深度神经网络的灵活性和参数*t*-SNE检索高维数据局部结构的能力整合在一个模型中,在人结直肠癌样本数据分析中表征了深层的肿瘤亚型特征。但*t*-SNE算法不能对新数据提供参数映射,方法转移效率低,这限制了其在大尺寸数据上的应用。

峰值拾取用于靶向提取具有高信号强度的质谱峰,再生成对应的图像来进行分析和可视化,从而降低原始数据的稀疏性和光谱维数,保留尽可能多的质荷比(*m/z*)特征信息。Abdelmoula等^[23]^基于全连接变分自编码器模型(variational autoencoder, VAE)建立了msiPL通用方法,通过对不同类型组织样本和质谱仪获取的MSI数据进行峰值学习(见图2),再使用概率生成算法从化合物原始数据的低维映射中生成与原始*m/z*等基本信息相关联的潜在五维编码特征,从而使分析独立于数据来源,在5种MSI数据集的肿瘤异质性研究中显示出良好的稳定性。为了避免不同研究人员进行参数优化时带入的潜在偏差,该团队又提出了massNet架构,直接基于高维数据学习特征,对MSI数据集进行快速高效分类,在没有前处理和峰值拾取的情况下可实现更高的准确度和显著的速度提升,避免过拟合导致测试集精度降低的问题^[24]^。

**图2:**
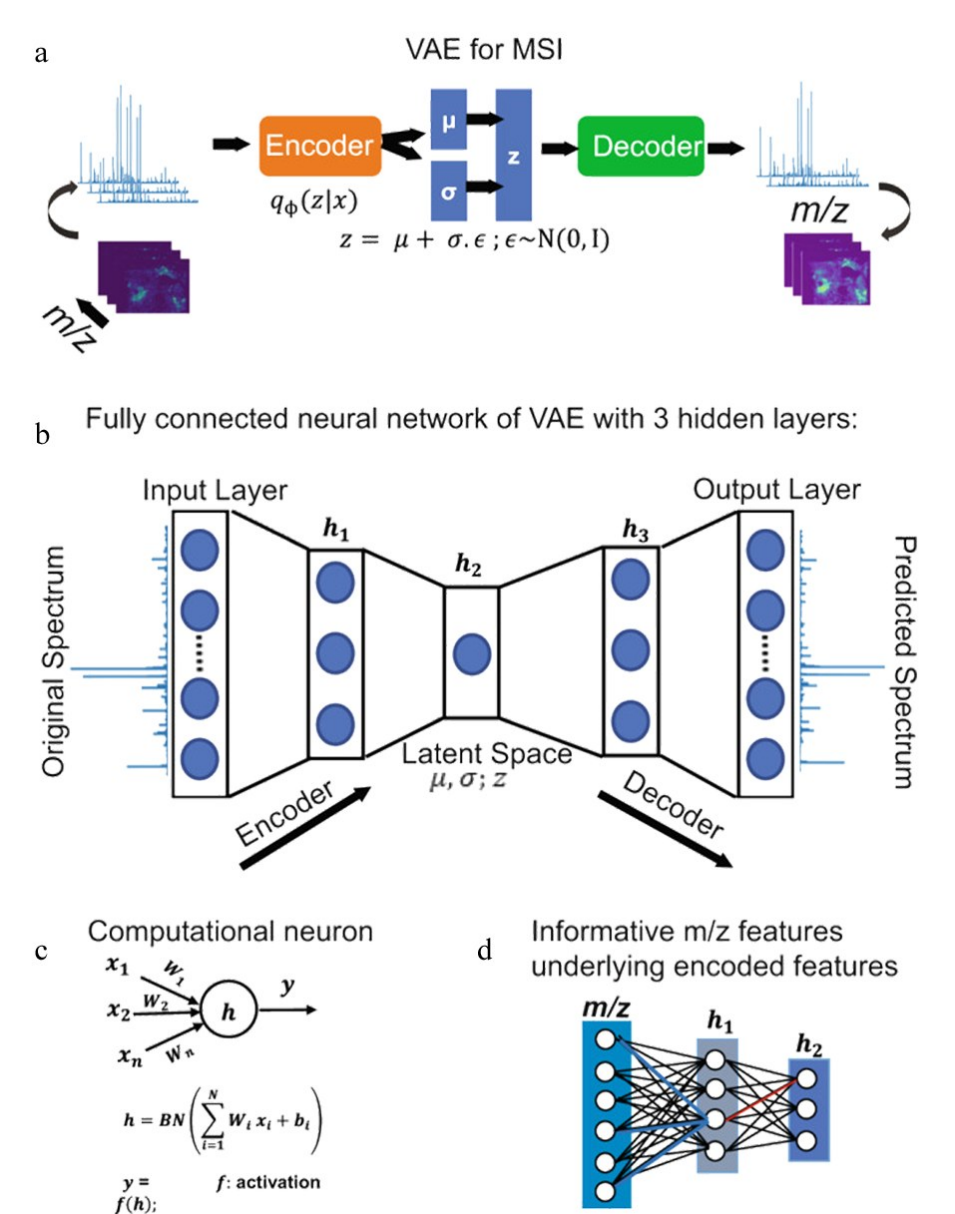
变分自编码器网络架构^[23]^

非线性DL对数据的降维能力固然强大,但其内部处理过程具有不可解释性,研究人员无法理解内部原理,因此模型的性能与可解释性之间需要充分权衡。Ma等^[25]^同时引入了可解释人工智能(explainable artificial intelligence, XAI)和通道选择两种策略以解决“黑匣子”不可解释的问题,并快速识别生物标志物。具体来讲,研究人员对残差网络(residual network, Res-Net)进行改进(Res-Net通过引入残差连接克服传统方法由于网络深度的增加造成模型训练效率严重降低的缺点),增加第一层卷积核数量来提高该层在整个模型的重要性,并在每次卷积时保留分类贡献排名较高的特征,提高关键特征挖掘能力,从而直接处理高维度MSI数据。作为验证,该团队在小鼠肌肉和人结直肠癌数据分析上比较了自建网络和7种传统ML方法,结果表明,自建网络在特征提取方面具有高准确度和稳定性,交叉验证准确率达到90%,远高于其他方法。

### 1.2 图像重构

图像重构是将按点采集的原始数据在数据预处理后转换为可视化的分布图像的过程。重构的目的是应用合适的数据处理、图像生成和可视化方法,将质谱数据转换为具有空间信息的图像,以直观展示样本中不同分子的分布情况。如何将大规模的数据集高效、高质量地构建成空间分布图像是一个重要挑战^[26]^。

为了构建组织完整图像,传统的数据采集方法是沿直线网格移动探头,通量低下,且离子源产生的高能粒子流会破坏样品。目前已经开发出替代的动态采样方法(dynamic sampling, DS),其原理是根据采集过程中获得的信息确定最佳扫描位置。Helminiak等^[27]^将DL与DS结合,提出DLADS(deep learning approach for dynamic sampling)策略,研究人员使用U-Net-CNN^[28]^生成初步图像,使用局部注意力模块根据图像的局部特征和上下文信息选择性调整采样区域,从而指导关键分子信息的数据采集。利用该策略对小鼠子宫和肾脏样本的研究结果表明,采集10%样本即可实现稳定、低误差的图像重构,同时使通量提高70%~80%。该团队的Hu等^[29]^进一步使用该策略对线扫描纳米喷雾解吸电喷雾电离(nanospray desorption electrospray ionization, nano-DESI)采样的适用性进行评估,通量提高2~3倍,显著扩大了MSI在生物研究中的应用范围。

三维(3D)MSI可以通过增加一个维度的方式来绘制复杂生物结构(如器官等)中的生物分子分布。然而,3D MSI是由一系列组织切片的二维(2D)MSI构建,其重构难度呈几何倍数增加。Li等^[30]^提出了3D MSI工作流程——DeepS(见图3a),通过建立3D稀疏采样网络(3D sparse sampling network, 3D-SSNet)来加速3D MSI分析。研究人员在两方面进行优化:①传统稀疏采样的像素设置为1×1,网络模型尺寸固定,当样本比例变化时无法处理对应像素,因此将采样单元定义为图像尺寸的公约数*k*; ②传统图像重构方法聚焦于推断未采样区域,而采样区域保持不变,因此可将单通道图像转换为三通道再进行平滑处理,改善不同区域间的过度。使用基于U-Net的3D-SSNet(见图3b)重建稀疏采样的组织切片,在采样比例设置为20%~30%的情况下实现与使用全采样MSI相近的结果。在阿尔兹海默症(AD)小鼠大脑的3D MSI研究中,该工作流程表现良好,通过迁移学习成功地应用于更多异质样本,如患有胶质母细胞瘤的小鼠大脑和小鼠肾脏等。

**图3:**
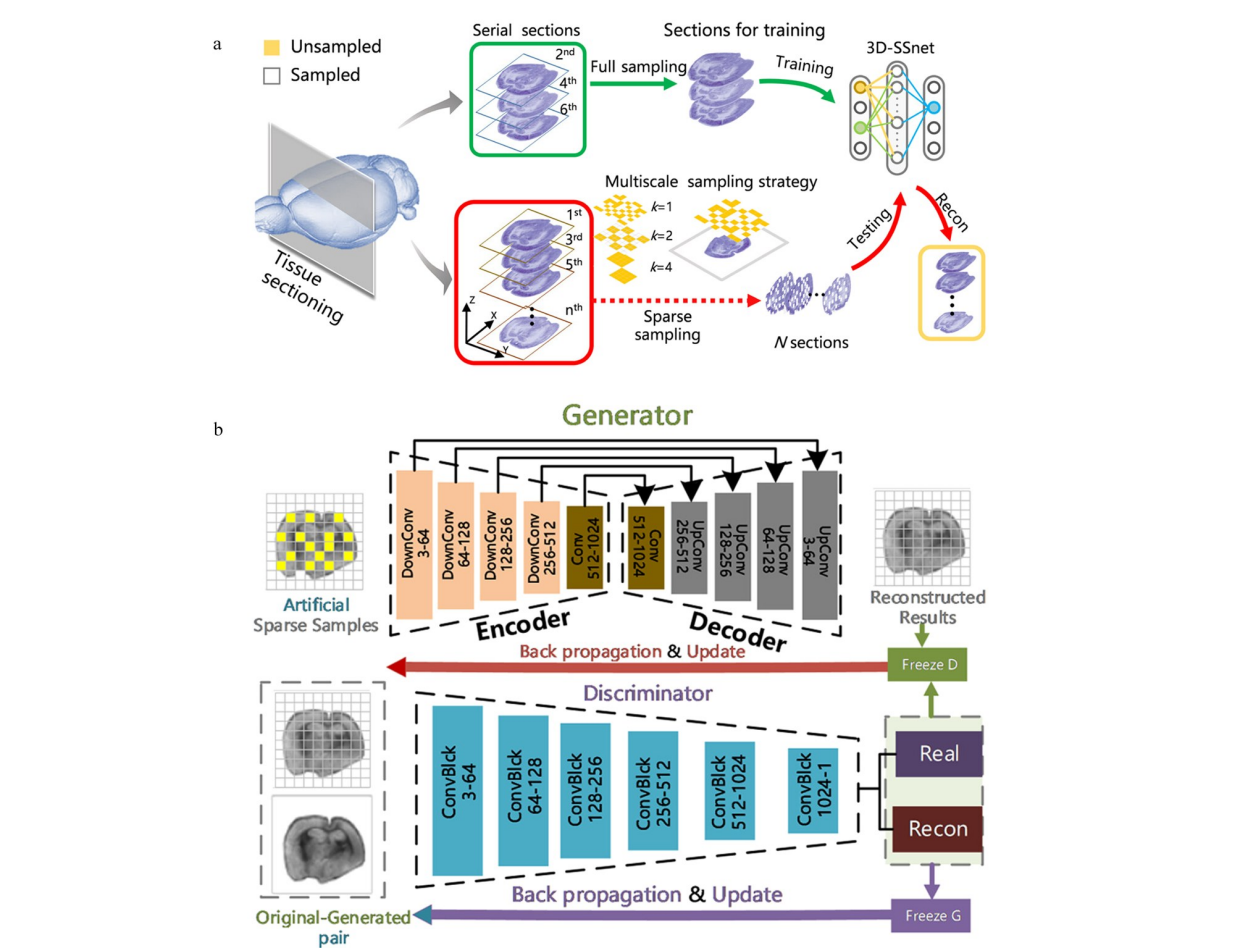
DeepS策略的流程图^[30]^

与MSI相比,液相色谱-质谱联用(liquid chromatography-mass spectrometry, LC-MS)在高通量表征代谢标志物时虽然无法获得代谢物的空间信息,但增加了保留时间(retention time, RT)维度的分离。Shen等^[31]^提出了伪质谱成像(pseudo-MSI)概念,即将LC-MS数据转换为图像进行分析。研究人员首先自建数据转换器脚本将LC-MS原始数据的RT、*m/z*和响应强度组成图像,再基于视觉几何组(visual geometry group,VGG)的监督算法提取图像特征,无需单独注释。在预测孕妇胎龄的研究中,该方法的准确度和稳定性优于传统方法;子宫内膜癌的癌症诊断准确率达到98%,证明了方法的实用性。此外,对自定义RT漂移数据集的预测结果证明伪成像能克服传统方法的多种缺点(RT漂移、响应强度漂移)。然而,该方法将像素进行固定压缩,可能造成特征丢失,无法准确注释代谢物,使深度学习成为“黑匣子”, Wang等^[32]^开发了MetImage策略(见图4),以0.01 Da的区间将MS数据分割为图像块,30%的数据空白以“0”填充完整,从而编码为多通道图像,该策略不仅保留了完整的质谱特征,还提高了与DL的数据兼容性,能更好地结合Res-Net进行训练和学习。在食管鳞状细胞癌(ESCC)的临床筛查研究中,标志物的特异性优于LC-MS方法,具有更高的准确率和更低的假阳性率,成功地识别和解释了关键图像块中的代谢信息。

**图4:**
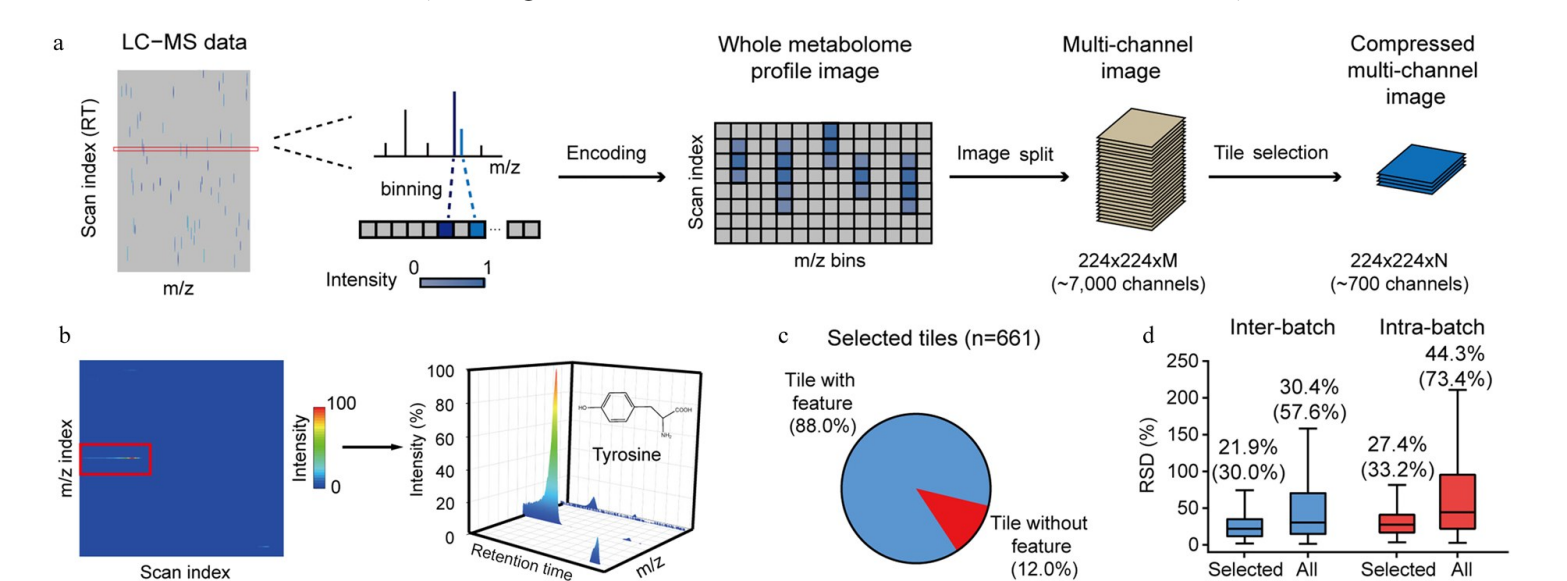
MetImage策略的流程图^[32]^

以上研究表明,将数据转换为图像进行分析有以下几个优点:①直观性:直接观察和比较特征的关系,易于理解和解释;②可视化:发现隐藏的重要信息、异常特征等;③数据压缩:转变数据存储方式,可能成为一种独特的临床诊断方法。

### 1.3 聚类分析

MSI图像包含样本分子分布信息,对具有差异分布的分子进行表征,并将相似规律的分子聚类为分子簇,对于分析生物标志物并阐明关键生物途径具有重要作用。图像聚类的过程包括特征提取、相似性度量、聚类算法和评估验证,通过提取特征表示,使用相似性度量方法衡量像素间的相似度,再采用聚类算法将相似的像素进行分组分析。MSI数据的高维复杂性和低信噪比特征为聚类分析带来挑战:①噪声、背景信号和数据稀疏性影响聚类识别的准确度;②样本组织的复杂组成和模糊边界增大区分难度;③对于无监督聚类的特征的解释与验证需要确保真实性。

#### 1.3.1 图像分割

手动分割图像困难且耗时,DL通过运用大规模的已标注图像数据进行训练,可以自动学习图像的特征和结构,从而实现高效准确的2D图像分割。

Behrmann等^[33]^首先将DL引入大规模肿瘤分类中,研究人员建立了基于Res-Net的IsotopeNet模型,结合了肽同位素峰组分布规律的局部编码特征。该模型从两类肿瘤的组织微阵列(TMA)的H&E图像的单位区域内监督学习人工注释特征,通过灵敏度分析预测与已报道肿瘤标志物灵敏度相近的特征,根据该特征进行肿瘤类型判断。该研究为MSI数据中的肿瘤分类领域做出了开创性贡献。为了避免产生过拟合,该团队的Janßen等^[34]^进一步进行了系统的参数优化,在非小细胞肺癌亚型分类分析中表现出较高的准确率,优于传统的LDA算法。

然而,用于模型训练的分类标签多由人工检查和注释产生,具有人工成本大和精确度差的缺点。因此,Guo等^[35]^引入了多实例学习(multiple instance learning, MIL)进行半监督分析,MIL将标签组合为实例包(instance bags),通过学习实例包之间的特征和关系来预测新的实例包标签,从而能够处理数据中的不完全标注信息。研究人员假设人工标记肿瘤区域是不完全准确的,仍包含肿瘤和非肿瘤的二元区域,根据LIME(local interpretable model-agnostic explanations)理论(通过生成对抗性样本扰动的方式,确定每个特征对于模型输出的影响程度)对标签和特征进行新预测和加权,从而实现对肿瘤亚组织的组织级精度注释。在人肾细胞癌和膀胱癌的区域划分研究中,相比于传统的支持向量机(SVM)算法,该方法整合多个特征来提高预测性能,实现更高的准确度。Isberg等^[36]^也评估了MIL在癌症诊断中的作用,准确率达到90%以上,但要使其常规应用于临床研究,还需要大量高质量的训练数据。

MSI数据实际上提供了更多的分子信息,存在无法从组织学或其他成像中发现的隐藏亚区,如果受到组织学数据或其他成像模式的监督,分割结果会产生偏差。Guo等^[37]^提出了分而治之(divide-and-conquer, dc)-DeepMSI策略(见图5),即采用两种无监督神经网络分别进行降维和聚类,对于降维,使用AE对数据进行前处理,对于聚类,使用2D CNN减小随机性,增加聚类稳定性。在空间连续MSI图像(小鼠胎儿)分析中,该模型准确地识别了12个器官,而传统k-means+*t*-SNE方法会过度聚类,SCiLS Lab软件由于采样特征选择机理而导致模糊聚类;在空间非连续图像(人乳腺癌MSI图像)分析中,癌区和癌旁子区分类清晰,证明了脂质类生物标志物具有显著的肿瘤异质性。该策略能降低整体数据分析的复杂性,在识别疾病进展和分子表型相关的亚区域方面表现良好。

**图5:**
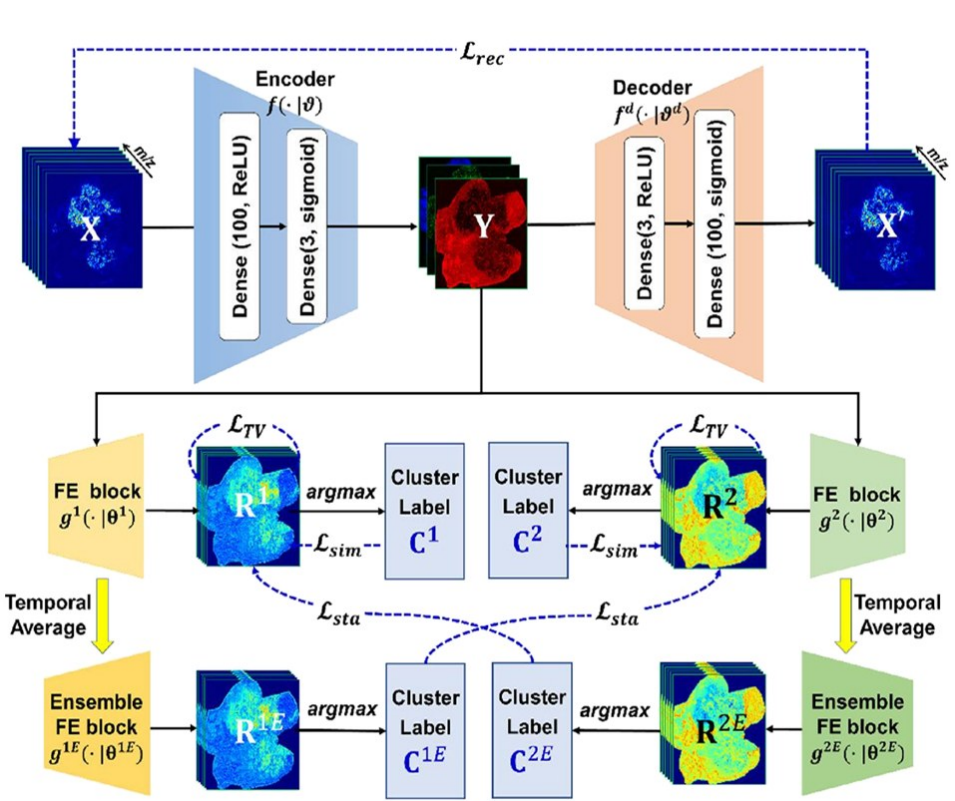
dc-DeepMSI网络架构^[37]^

#### 1.3.2 空间聚类

空间聚类是基于分子共定位或多通道图像融合的分析方式,其难点在于数据维度的增加以及分子种类的复杂性和干扰信号的负面影响等因素加大了数据分析的难度,因此需要强大的算法以提高分析的准确性和可靠性。

通过离子图像之间空间相似性的度量进行空间聚类、分割出感兴趣空间区域是可行性较高的方法,已有多种量化共定位的度量,如Pearson相关系数、余弦值等,但尚未有研究综合评估这些度量的可靠性。Ovchinnikova等^[38]^建立了机器学习方法量化空间共定位(co-localization machine learning, ColocML),使用Xception网络^[39]^(Xception网络通过模块堆叠和分类卷积来提高特征提取效率,具有更高的图像识别和分类性能)建立半监督深度学习Pi模型,在METASPACE数据集中公认的高质量数据上测试,结果表明该模型与中值阈值化后的余弦相似度是最佳度量方法。基于全局的度量方法容易忽略图像的局部相关性,Zhang等^[40]^在Ovchinnikova等的基础上采用无监督方式训练模型,将离子图像分割为自定义大小的模块进行特征学习(见图6),从而提高对局部特征的获取,生成的神经离子图像作为后续聚类分析的输入,性能得到提高。

**图6:**
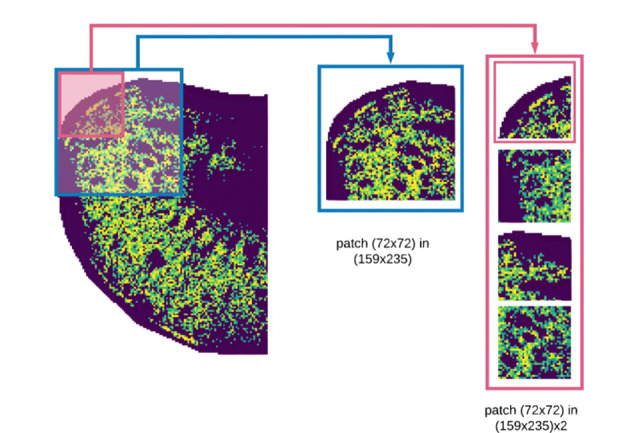
采样斑块划分示意图^[40]^

传统的计算方法是基于图像矢量的相似性度量来确定分子共定位,无法关联高级空间特征,因此降低了对具有相似定位但不同对比度的空间模式的泛化能力。Hu等^[41]^通过引入对比学习(contrastive learning)建立了基于分子共定位的聚类方法,对比学习是一种自监督学习方法,通过将正样本(相似的样本)与负样本(不相似的样本)进行比较,最大化正样本之间的相似性,并最小化正样本与负样本之间的相似性。研究人员将预训练的EfficientNet^[42]^(EfficientNet网络通过复合缩放,优化参数量的方式提高计算效率,比Res-Net、Xception等网络提高一个数量级)作为基础CNN,通过数据增强处理提高模型泛化能力。在完全注释小鼠子宫和未注释小鼠脑样本数据集的聚类研究中,对神经网络的再训练将分类准确率提高了20%,从而实现了高准确度的无注释条件下的分子共定位分析。

### 1.4 多模态融合

多种成像技术(如组织学成像、MSI等)已广泛应用于复杂生物样品的研究,每种成像技术在不同的长度尺度和空间分辨率下提供互补的、偶尔重叠的信息。多模态融合是将不同成像技术表征的目标特征进行传递和融合分析,提供对目标的全面解释。以MSI数据为主导的多模态融合具有一些挑战和难点,主要包括以下几个方面:①数据融合:各种图像间存在差异,如成像区域、分辨率、噪声性质等,需要进行有效的数据对齐,以确保图像在空间和化学信息方面的一致性;②数据解释:多模态融合会引入更复杂的信息,需开发合适的方法提取和解释有用信息;③统一和标准化:制定一致的数据格式是不同成像可比性的基础,需要建立共享平台来实现不同技术的兼容。

在MSI和组织学成像的融合分析方面,通常采用监督学习方法实现准确注释,Race等^[43]^提出了一种通用多模态配准流程,使用预训练的DenseNet学习H&E图像的肿瘤分类特征,生成带注释的灰度图像,再与同一切片MSI数据的聚类结果进行交叉转移,可实现精准的ROI划分(见图7A)。Janßen等^[44]^提出了双峰肿瘤分型方法,首先应用U-Net将H&E图像划分肿瘤和非肿瘤区域,使用先前开发的IsotopeNet从划分的肿瘤区域中学习和训练特征,再配准到MSI数据,实现肿瘤亚型诊断。Haque等^[45]^采用基于Res-Net的DL方法研究前列腺癌诊断,从成对的H&E和MSI成像中学习癌变区域特征和空间配准,再直接对H&E图像进行癌症判断,使化学特征与组织解剖结构相关联,准确率达到80%。

**图7:**
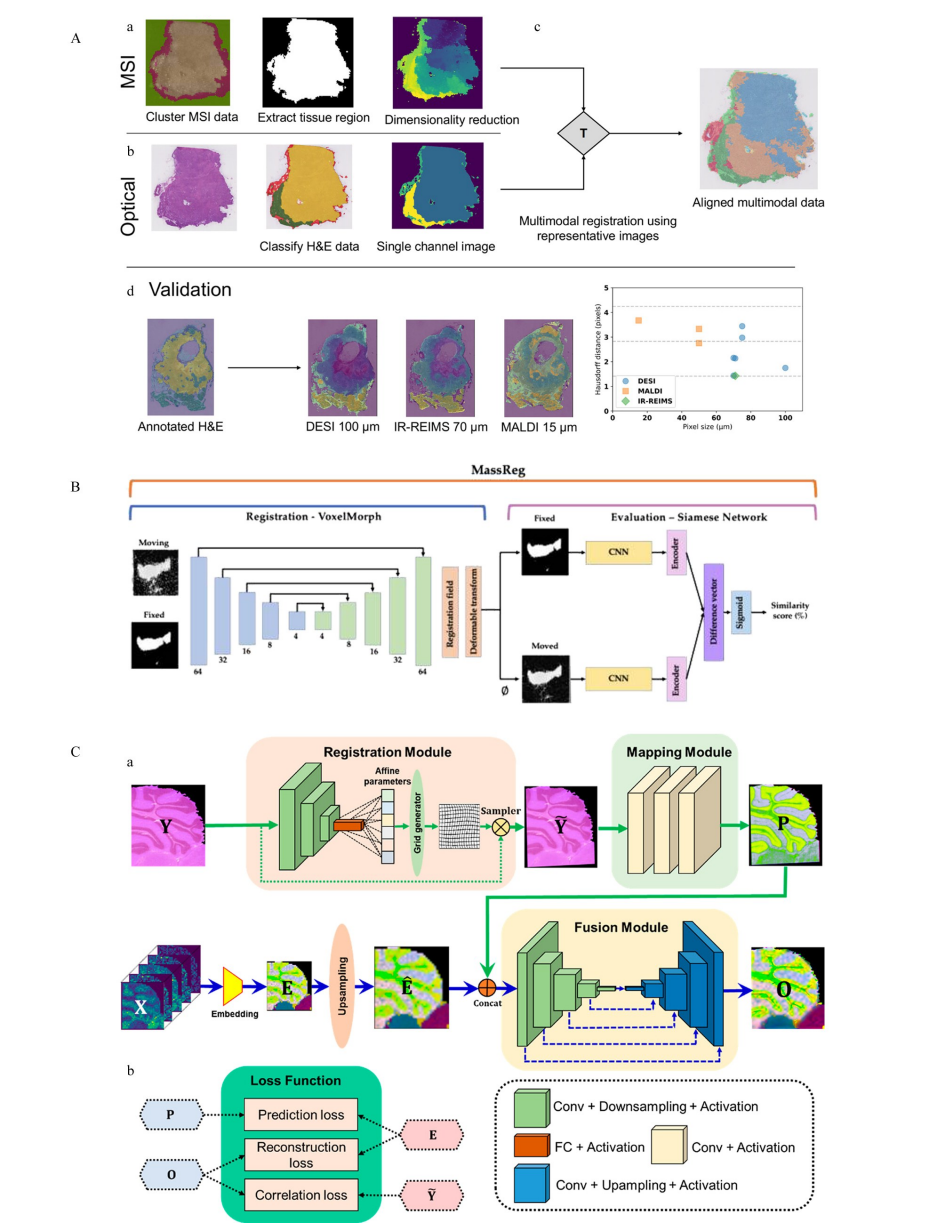
3种用于多模态融合的深度学习策略

然而,监督学习方法依赖于大量经过精准注释的数据集,因此不能得到广泛应用。Connolly等^[46]^提出了MassReg策略(见图7B),该策略包括配准和评估两个部分:配准采用VoxelMorph方法(一种非刚性图像配准方法^[47]^),将H&E和MSI两种图像作为输入并输出转换字段进行配准;采用Siamese无监督可变形网络进行配准输出评估,并使用模拟和真实图像多次迭代数据训练神经网络。在前列腺活检组织的DESI和组织学图像对的配准上证明了该策略的实用性。图像配准后,划定与不同解剖或病理标记的组织区域相对应的ROI是必不可少的,Guo等^[48]^使用预训练的深度卷积神经网络(deep convolutional neural network, DCNN)将H&E图像的颜色和形态共同编码为组织学特征(HFs),储存在高维数据立方体中,通过NMF算法提取区域特征,再手动正交配准,结合聚类算法实现ROI划分。在小鼠肾脏和肾脏肿瘤样本分析中,多模态融合分析证实的ROI区域与实际区域非常吻合。

前述方法多侧重于将MSI图像中的像素强度与其他模态图像中相应位置的像素强度进行匹配,很少考虑全局空间结构的相似性。而且,图像配准和数据融合通常被视为两个独立的模块,因此其优化目标的偏差可能会导致重建结果的偏差。超分辨率算法是基于ML和DL进行图像恢复和提高图像分辨率的技术,在计算机视觉领域具有重要应用^[49]^。Guo等^[50]^提出了一种多模态融合策略DeepFERE(见图7C),该策略引入空间变换网络(spatial transformation network, STN)实现自动配准,使用U-Net从H&E图像预测高分辨MSI图像,将预测图像通过二次插值法嵌入到LR-MSI,从而生成重建HR-MSI,该模型在视觉检测和定量评价两方面都能够产生具有丰富化学信息和详细结构的高分辨率重建图像,在小鼠脑组织图像配准上产生最佳效果。Liao等^[51]^提出了MOSR (MSI from optical super-resolution)策略,该策略采用基于增强型生成对抗网络(ESRGAN)的超分辨率算法,在大型参考光谱数据库中进行参数训练,迁移到小样本量HSR-MSI图像模型中构建映射关系,从而精准预测出批量HSR-MSI图像,迁移学习使该模型有更高的泛化能力和准确度。该模型可实现20 μm级别研究,但受到训练集中光谱分辨率的限制,其应用能力需要进一步评估和拓展。

基于DL开发新的算法和策略来解决多模态融合存在的问题和挑战,实现更精准和高效的分析,其应用效果远优于传统方法。未来,多模态融合将逐渐普遍应用,囊括更多的技术,解决更复杂的生物学和医学问题。

深度学习在MSI数据分析各个阶段的应用方法总结见表1,研究人员通过采用不同的神经网络、算法模式等针对性解决不同数据分析阶段存在的问题,如在数据降维和多模态融合方面,侧重于无监督模式,在提高分析效率的同时能保留数据的真实性和可解释性;在聚类分析方面,为了引入真实结果预训练网络模型来提高分析准确度,侧重于监督和半监督模式。相较于传统ML方法,深度学习在算法优越性、分析效率和结果准确性等方面优势显著,促进MSI在生物研究中发挥重要作用。

**表1:** 用于MSI数据分析的深度学习方法总结

Function	Neural networks	Model	Sample/ Dataset	Acquisition methods	Description	Refs.
Data prepro- cessing	AE	unsuper- vised	rat brain	MALDI	the first exploration of deep learning in MSI data dimensionality reduction	[21]
	AE	unsuper- vised	human colorectal carcinoma	DESI	unsupervised parameterization dimensionality reduction method established by combining DNN and *t*-SNE	[22]
	VAE	unsuper- vised	METASPACE	MALDI, DESI, FT-ICR	fully connected VAE for unsupervised peak learning of different instrument/sample datasets	[23]
	massNet, VAE	-	mouse brain spongio- blastoma	MALDI	scalable massNet framework for directly learning of features from high-dimensional data	[24]
	Res-Net	-	mouse muscle, human colorectal carcinoma	MALDI	Res-Net model based on channel selection to directly extract and characterize features	[25]
Image recon- struction	U-Net, MLP	supervised	mouse uterus, kidney	nano-DESI	sparse dynamic sampling planning and image reconstruction by U-Net CNN	[27, 29]
	U-Net-GAN	-	rat brain spongioblas- toma, kidney	MALDI	customize sampling unit and adversarial learning to optimize and improve accuracy of image reconstruction	[30]
	VGG	supervised	maternal plasma	ESI	irstly proposed to construct pseudo-images by using multi-dimensional information of LC-MS	[31]
	Res-Net	-	human esophagus squamous cell carcino- ma serum	ESI	image blocks based custom multi-channel approach to optimize pseudo-imaging accuracy	[32]
Image segmentation	IsotopeNet, Res-Net	supervised	pancreatic/squamous cell carcinoma, lung/ pancreatic tumor	MALDI	the first application of Res-Net in feature extraction and ROI labeling of MSI	[33]
	IsotopeNet	-	human non-small cell lung cancer	MALDI	a tumor classification system integrating DL and LDA classification algorithms	[34]
	MIL-CNN	semi-super- vised	human renal cell carci- noma, bladder cancer	MALDI, DESI	semi-supervised MXL framework for tissue level annotation to realize tumor sub-tissue labeling and classification	[35]
	MIL-CNN	semi-super- vised	human breast cancer	DESI	application of MIL in cancer diagnosis with high accuracy	[36]
	AE, CNN	unsuper- vised	mouse fetus, human breast cancer	MALDI	dc-DeepMSI model and DL algorithm based data reduction and feature clustering	[37]
Spatial clustering	Xception	semi-super- vised	METASPACE	MALDI	Xception network based semi-supervised Pi model showed best molecular colocalization ability	[38]
	Xception, ANN	unsuper- vised	human lymph nodes, mouse kidney	MALDI	Xception network based neural ion channel realized more accurate spatial clustering	[40]
	EffcientNet	self-supervised	METASPACE	MALDI	contrast learning based CNN model realized unannotated molecular colocalization	[41]
Multimodal fusion	DenseNet	supervised	mouse adenocarcino- ma	DESI	DenseNet based features annotation for automated tumor ROI division	[43]
	IsotopeNet, U-Net	-	human non-small cell lung cancer	MALDI	combining of U-Net and IsotopeNet for ROI analysis and tumor annotation of MSI	[44]
	Res-Net	semi-super- vised	human prostate	MALDI	similarity learning between H&E imaging and MSI data by Res-Net	[45]
	SiameseNet, U-Net	unsuper- vised	human prostate	DESI	MassReg model containing U-Net annotation learning and SiameseNet output	[46]
	DCNN	unsuper- vised	mouse kidney	DESI	the multimodal fusion strategy realized the custom feature extracting and matching through DCNN	[48]
	CNN	unsuper- vised	mouse brain, human liver cancer	DESI	spatial transformation network based DeepFERE model for high-resolution images construction	[50]
	GAN	-	mouse brain	MALDI	MOSR model by combining multiple networks to build the mapping relationship and predict ultra-high-resolution images	[51]

-: not given in the reference. AE: autoencoder; VAE: variational autoencoder; CNN: conventional neural network; Res-Net: residual network; GAN: generative adversarial network; VGG: visual geometry group; MIL: multiple instance learning; MALDI: matrix-assisted laser desorption ionization; DESI: desorption electrospray ionization; DL: deep learning; LDA: linear discriminant analysis.

## 2 结合深度学习的质谱成像技术在肿瘤诊断等研究中的应用

肿瘤是一类高度异质性的疾病,不同类型的肿瘤及不同部位的肿瘤在分子水平上存在巨大差异。对肿瘤的发生、发展、治疗和预防等方面进行系统、科学的研究,深入了解肿瘤的生物学特性、分子机制以及与宿主的相互作用,对推动肿瘤的早期诊断、精准治疗和预防控制具有重要意义。

近年来,组织学技术的快速发展使生物分子的深入分析成为可能,但仍未克服耗时、耗力、主观性强、分辨率和全面性不足的问题。基于质谱成像的空间分辨组学方法在高通量获取分子定性特征的同时还保留了位置信息,可直接观察肿瘤组织中大量的代谢物、蛋白质^[52]^等分子,了解其在空间上的异质性^[53]^,不依赖特定分子的先验知识,为肿瘤分类、分级、药物作用评估和生物标志物发现提供支持^[54]^。强大的深度学习算法能处理更复杂的数据,发现更深层和更准确的特征^[55]^。

非小细胞肺癌(non-small cell lung cancer, NSCLC)是最常见的肺癌类型,约占所有肺癌的80%~85%。NSCLC主要包括两个最常见的亚型:腺癌(adenocarcinoma, ADC)和鳞癌(adenocarcinoma, SqCC);在组织学上,样本通常表现为混合型肿瘤,且不同部位的亚型也存在肿瘤异质性,造成人工诊断的时间成本消耗和准确率低下,约20%的样本无法被正确分类^[56]^。Kriegsmann等^[57]^通过MALDI-MSI获取TMA样本的分子分布特征,建立了含300个特征的LDA分类模型,对118例样本的诊断准确率达到99.1%, 7个主成分具有明显差异。Boskamp等^[58]^研究表明基于图谱特征的LDA算法的准确率高于基于*m/z*特征的LDA算法。端到端学习(在学习过程中不需明确给出不同模块或阶段的功能,中间过程不需要人工干预,一步学习特征并分类)是一种有潜力的替代方式,Behrmann等^[33]^初步引入神经网络进行分类研究,在相同数据集上提取到已报道的特征,对NSCLC的分类准确率与Boskamp等相近。近期,该团队的Janßen等^[34]^进一步优化了神经网络参数,测试集准确率达到100%,还证明了LDA方法的特征积累会导致过拟合。同年,Janßen等^[44]^改变了基础网络架构算法并扩大了训练集包含的癌症种类,使模型不再局限于识别固定特征,在完整癌症切片样本上的分类准确率为87.5%(14/16)。该团队的一系列研究显著提高了非小细胞肺癌的亚型诊断准确率,有助于病理学家的决策,为人工智能转化为临床常规方法用于肿瘤诊断起到了积极的推动作用。

前列腺癌(prostatic cancer, PC)是一种组织高度异质性癌症,是男性最常见的癌症之一,发病率约为1/8。Connolly等^[46]^开发了基于深度学习的注释转移方法,在人前列腺癌样本的MSI图像中可以成功识别组织的轮廓,同时剔除大部分背景噪音。Haque等^[45]^基于深度学习提取已表征的MSI特征,对组织学成像进行注释,从而区分癌区和非癌区(人工注释误差和其他背景区域),准确率达到80%,该研究为开发更准确的预测患者PC并改善患者护理提供了数据支持。

其他领域也需要深度学习进行辅助分析,例如,在脑科学研究中,Yamada等^[59]^使用CNN从小鼠大脑切片的训练脑区提取特征,实现对脑区域的组织学分割和分类;Xie等^[60]^建立了MEISTER框架,使用深度自编码器(deep autoencoder, DAE)将单细胞细胞分型与化合物空间分布配准,创建全脑单细胞生化信息图谱,表征了具有高度区域异质性的脂质类生物标志物。

总之,结合深度学习的质谱成像方法在肿瘤疾病研究以及其他生物医学研究中发挥重要作用,为肿瘤诊断、治疗和疾病研究提供了新的视角和手段。随着分析策略的进一步发展和数据的积累,这一领域将持续推动精准医学的发展。

## 3 总结与展望

近年来,深度学习在质谱成像数据分析中的应用研究取得了显著进展,在数据的前处理、特征提取和图像解释等方面展现出了优越性能。深度学习方法能有效处理质谱成像数据中的噪声和伪迹,提高数据质量和准确性;在特征提取方面能够自动学习数据中的特征,以矩阵、图像等方式表现,并通过特征融合和选择提高质谱成像图像的可辨识性和解析度;在图像解释方面能够识别并定位图像中的重要结构和区域,实现对质谱成像数据的快速解析与分析。然而,仍然存在一些挑战和需要进一步解决的问题。质谱成像数据通常具有高维度和巨大的数据量,需要更高效和可扩展的深度学习模型和算法;模型的可解释性仍然是一个关键问题,在解释深度学习模型输出结果的同时,需更好地揭示质谱成像数据背后的生物学意义;为了能够更好地应用和推广深度学习方法,有必要建立更完备的数据集和开源工具包。未来的发展将集中在模型的优化、可解释性的提高以及与其他技术的融合,扩展质谱成像数据分析的研究领域和应用前景,为质谱成像在生命科学和医学领域的应用提供更丰富和可靠的数据分析工具。

## References

[1] Luxembourg S L, Mize T H, McDonnell L, et al. Anal Chem, 2004, 76(18): 5339 15362890 10.1021/ac049692q

[2] Buchberger A R, DeLaney K, Johnson J, et al. Anal Chem, 2017, 90(1): 240 29155564 10.1021/acs.analchem.7b04733 PMC5959842

[3] Mellinger A L, Muddiman D C, Gamcsik M P. J Proteome Res, 2022, 21(8): 1800 35749637 10.1021/acs.jproteome.2c00220 PMC12551641

[4] Palmer A, Phapale P, Chernyavsky I, et al. Nat Methods, 2017, 14(1): 57 27842059 10.1038/nmeth.4072

[5] Duhamel M, Drelich L, Wisztorski M, et al. Nat Commun, 2022, 13(1): 6665 36333286 10.1038/s41467-022-34208-6 PMC9636229

[6] Li F, Luo Q. Chinese Journal of Chromatography, 2024, 42(2): 150 38374595 10.3724/SP.J.1123.2023.11005 PMC10877477

[7] Hou J J, Zhang Z J, Wu W Y, et al. Acta Pharmacol Sin, 2022, 43(12): 3096 36229602 10.1038/s41401-022-00990-8 PMC9712638

[8] Randall E C, Emdal K B, Laramy J K, et al. Nat Commun, 2018, 9(1): 4904 30464169 10.1038/s41467-018-07334-3 PMC6249307

[9] Dong Y, Aharoni A. Nat Pro Rep, 2022, 39(7): 1510 10.1039/d2np00011c 35735199

[10] Zou Y, Tang W, Li B, et al. Int J Food Microbiol, 2022, 371: 109675 35427956 10.1016/j.ijfoodmicro.2022.109675

[11] Guo X, Wang X, Tian C, et al. Talanta, 2023, 264: 124721 37271004 10.1016/j.talanta.2023.124721

[12] Kim J Y, Seo E S, Kim H, et al. Nat Commun, 2017, 8(1): 2113 29235455 10.1038/s41467-017-02216-6 PMC5727394

[13] Kompauer M, Heiles S, Spengler B. Nat Methods, 2017, 14(1): 90 27842060 10.1038/nmeth.4071

[14] Porta Siegel T, Hamm G, Bunch J, et al. Mol Imaging Biol, 2018, 20(6): 888 30167993 10.1007/s11307-018-1267-y PMC6244545

[15] Coskun A F, Han G, Ganesh S, et al. Nat Commun, 2021, 12(1): 789 33542220 10.1038/s41467-020-20753-5 PMC7862654

[16] LeCun Y, Bengio Y, Hinton G. Nature, 2015, 521(7553): 436 26017442 10.1038/nature14539

[17] He J, Huang L, Tian R, et al. Anal Chim Acta, 2018, 1015: 50 29530251 10.1016/j.aca.2018.02.030

[18] Huang L, Mao X, Sun C, et al. Anal Chim Acta, 2019, 1077: 183 31307708 10.1016/j.aca.2019.05.068

[19] Hu H, Laskin J. Adv Sci (Weinh), 2022, 9(34): e2203339 36253139 10.1002/advs.202203339 PMC9731724

[20] Song X, Zang Q, Zhang J, et al. Anal Chem, 2023, 95(17): 6775 37021399 10.1021/acs.analchem.2c01723

[21] Thomas S A, Race A M, Steven R T, et al. IEEE SSCI, 2016: 1

[22] Inglese P, McKenzie J S, Mroz A, et al. Chem Sci, 2017, 8(5): 3500 28507724 10.1039/c6sc03738k PMC5418631

[23] Abdelmoula W M, Lopez B G, Randall E C, et al. Nat Commun, 2021, 12(1): 5544 34545087 10.1038/s41467-021-25744-8 PMC8452737

[24] Abdelmoula W M, Stopka S A, Randall E C, et al. Bioinformatics, 2022, 38(7): 2015 35040929 10.1093/bioinformatics/btac032 PMC8963284

[25] Ma W, Luo L, Liang K, et al. Anal Bioanal Chem, 2023, 415(14): 2819 37083759 10.1007/s00216-023-04694-8

[26] Belthangady C, Royer L A. Nat Methods, 2019, 16(12): 1215 31285623 10.1038/s41592-019-0458-z

[27] Helminiak D, Hu H, Laskin J, et al. Electron Imaging, 2021, 33(15): 2901 10.2352/issn.2470-1173.2021.15.coimg-290 PMC8553253 34722959

[28] Ronneberger O, Fischer P, Brox T. MICCAI. (2015-05-18). http://lmb.informatik.uni-freiburg.de/people/ronneber/u-net http://lmb.informatik.uni-freiburg.de/people/ronneber/u-net

[29] Hu H, Helminiak D, Yang M, et al. ACS Meas Sci Au, 2022, 2(5): 466 36281292 10.1021/acsmeasuresciau.2c00031 PMC9585637

[30] Li D, Qian Y, Yao H, et al. Anal Chem, 2023, 95(29): 10879 37427961 10.1021/acs.analchem.2c05785

[31] Shen X, Shao W, Wang C, et al. Brief Bioinform, 2022, 23(5): bbac331 35947990 10.1093/bib/bbac331

[32] Wang H, Yin Y, Zhu Z J. Anal Chem, 2023, 95(16): 6533 37042095 10.1021/acs.analchem.2c05079

[33] Behrmann J, Etmann C, Boskamp T, et al. Bioinformatics, 2018, 34(7): 1215 29126286 10.1093/bioinformatics/btx724

[34] Janßen C, Boskamp T, Hauberg-Lotte L, et al. Proteom Clin Appl, 2022, 16(4): e2100068 10.1002/prca.202100068 35238465

[35] Guo D, Föll M C, Volkmann V, et al. Bioinformatics, 2020, 36: i300 32657378 10.1093/bioinformatics/btaa436 PMC7355295

[36] Isberg O G, Giunchiglia V, McKenzie J S, et al. Metabolites, 2022, 12(5): 455 35629959 10.3390/metabo12050455 PMC9143055

[37] Guo L, Dong J, Xu X, et al. Anal Chem, 2023, 95(3): 1924 36633187 10.1021/acs.analchem.2c04045 PMC9878502

[38] Ovchinnikova K, Stuart L, Rakhlin A, et al. Bioinformatics, 2020, 36(10): 3215 32049317 10.1093/bioinformatics/btaa085 PMC7214035

[39] Chollet F. 2017 IEEE Conference on Computer Vision and Pattern Recognition. Honolulu: IEEE, 2017: 1251

[40] Zhang W, Claesen M, Moerman T, et al. Anal Bioanal Chem, 2021, 413(10): 2803 33646352 10.1007/s00216-021-03179-w PMC8007517

[41] Hu H, Bindu J P, Laskin J. Chem Sci, 2022, 13(1): 90 10.1039/d1sc04077d PMC8694357 35059155

[42] Tan M, Li Q. ICML, 2019, 97: 6105

[43] Race A M, Sutton D, Hamm G, et al. Anal Chem, 2021, 93(6): 3061 33534548 10.1021/acs.analchem.0c02726

[44] Janßen C, Boskamp T, Le'Clerc Arrastia J, et al. Cancers (Basel), 2022, 14(24): 6181 36551667

[45] Haque M I U, Mukherjee D, Stopka S A, et al. J Am Soc Mass Spectr, 2023, 34(2): 227 10.1021/jasms.2c00254 PMC10479534 36625762

[46] Connolly L, Jamzad A, Kaufmann M, et al. J Imaging, 2021, 7(10): 203 34677289 10.3390/jimaging7100203 PMC8539093

[47] Balakrishnan G, Zhao A, Sabuncu M R, et al. IEEE Trans Med Imaging, 2019: 1788 10.1109/TMI.2019.2897538 30716034

[48] Guo A, Chen Z, Li F, et al. GigaScience, 2022, 12: giad021 37039115 10.1093/gigascience/giad021 PMC10087011

[49] Dong C, Loy C C, He K, et al. IEEE PAMI, 2016, 38: 295 10.1109/TPAMI.2015.2439281 26761735

[50] Guo L, Zhu J, Wang K, et al. Anal Chem, 2023, 95(25): 9714 37296503 10.1021/acs.analchem.3c02002

[51] Liao T, Ren Z, Chai Z, et al. Nat Mach Intell, 2023, 5(6): 656

[52] Guo G, Papanicolaou M, Demarais N J, et al. Nat Commun, 2021, 12(1): 3241 34050164 10.1038/s41467-021-23461-w PMC8163805

[53] Sun C, Wang A, Zhou Y, et al. Nat Commun, 2023, 14(1): 2692 37164975 10.1038/s41467-023-38360-5 PMC10172194

[54] Zhou Y, Jiang X, Wang X, et al. J Pharm Anal, 2023, 13(8): 851 37719191 10.1016/j.jpha.2023.07.003 PMC10499658

[55] Kleppe A, Skrede O J, De Raedt S, et al. Nat Rev Cancer, 2021, 21(3): 199 33514930 10.1038/s41568-020-00327-9

[56] Mukhopadhyay S, Katzenstein A L. Am J Surg Pathol, 2011, 35(1): 15 21164283 10.1097/PAS.0b013e3182036d05

[57] Kriegsmann M, Casadonte R, Kriegsmann J, et al. Mol Cell Proteomics, 2016, 15(10): 3081 27473201 10.1074/mcp.M115.057513 PMC5054336

[58] Boskamp T, Lachmund D, Oetjen J, et al. Biochim Biophys Acta Proteins Proteom, 2017, 1865(7): 916 27836618 10.1016/j.bbapap.2016.11.003

[59] Yamada H, Xu L, Eto F, et al. J Am Soc Mass Spectr, 2022, 33(9): 1607 10.1021/jasms.2c00080 35881989

[60] Xie Y R, Castro D C, Rubakhin S S, et al. Nat Methods, 2024, 21(3): 521 38366241 10.1038/s41592-024-02171-3 PMC10927565

